# St. George's Hospital

**Published:** 1902-04-26

**Authors:** 


### ST. GEORGE'S HOSPITAL.

The annual meeting at this hospital was a formal one. A bare quorum of Governors sat round the board-room table on Friday, April 18th, and got through the business in twenty minutes, but there was nothing of interest except the chairman's remarks on dentistry. He wanted to know if we expected more of our teeth than we used to do, and he evidently did not quite see what the dental surgeon wanted with an assistant.

However the appointment was made.

The laws were then altered to admit of the appointment of a physician-anæsthetist. The member of the board who proposed the appointment said that of late years it had become almost a necessity that the man engaged in giving anæsthetics should be engaged in that work only. Dr. Hewett, he said, was a recognised authority on the matter, and he was willing to join the staff. His services would be, as others were, perfectly gratuitous; the matter had been before the medical school committee for some time, and they unanimously recognised the necessity of such an appointment. The following law was enacted:—

### The Physician-Anæsthetist.

"The physician-anæsthetist shall be a graduate in medicine or surgery of a British university, or a Fellow or Member of the Royal College of Physicians of London, or a Fellow of the Royal College of Surgeons of England, who shall be engaged exclusively in the practice of anæsthetics. He shall exercise such supervision over the administration of anæsthetics in the hospital as the Medical School Committee shall from time to time arrange with the approbation of the weekly board."

While the business went on, voting by ballot took place; this was for "a committee consisting of twenty-one governors in addition to certain officers of the hospital, to form a committee to fill vacancies in any of the offices mentioned in Law 67 which may occur during the ensuing twelve months."

The court was both general and special, and among the special business was the adoption of the annual report and accounts, which were taken as read. A copy of the latter had been sent to all the governors, with a notice of the court. The meeting, which opened with prayer by the chaplain at a few minutes past one o'clock, was over by 1.30,

### The Year's Work.

This is a year of special appeal, as anyone who passes Hyde Park Corner is likely to be aware; large placards set forth the comparison of income and expenditure, and the name of the hospital, and the fact of its support by voluntary contributions, have been picked out in red paint. There is, further, an advertisement that seats to view the Coronation procession may be had within; three wards at £300 each have already been taken, and altogether the sales appear to be going well. Among other distinguished spectators will be the Duke of Norfolk, who has taken a ward.

The report opens with the statement that the Weekly Board were much concerned when the King said he could not undertake the office of President, which was held by the late Queen Victoria, but that their anxiety lest Royal patronage should be accorded in a less degree than hitherto was removed when the Prince of Wales accepted the office. The Prince and Princess visited the hospital early in the present year, going through the wards and speaking to most of the patients. They thought the bedsteads and furniture needed renewal, and that electric light should be supplied. They offered to give bedsteads for one ward, and, the offer being gratefully accepted, a committee was appointed to consider the questions brought to the notice of the board, who, however, had them under consideration already. Princess Christian has also been to see the hospital, and was particularly pleased with the nurses' new quarters.

The work of the hospital has been regular and uninterrupted; 4,988 patients were treated, the daily average number being 299·63. This low average is due to the fact that the Nurses' Home had to be repaired and re-painted, and so three wards were closed for nearly eight months to accommodate the nurses. The mean residence was 21·91 days. There are 351 beds. There were 31,843 out-patients, all of whom were treated without letters; 3,360 in-patients also required no letters, the urgency of the case being sufficient recommendation. Only 799 patients were sent to the Convalescent Hospital at Wimbledon, as some of the ceilings fell in and others were dangerous.

Annual subscriptions were £5,438; donations, £3,345; Hospital Sunday Fund, £1,937; Saturday Fund, £358. There is a decrease of £300 in subscriptions, and an increase of £183 in donations, compared with 1900. King Edward's Fund gave again £1,000 as a special grant, and the Board were disappointed that it was not more. They drew the attention of the Visitors of the Fund to the old bedsteads and other furniture, which they hoped to replace by some of more modern construction, and they hoped that the Council would have helped them with funds for refurnishing. The total ordinary income was £27,686, the expenditure £40,840. The extraordinary income was £5,739, the extraordinary expenditure £4,555. Stock, during the last ten years, has been sold out to the amount of £90,639; of this only £38,251 has been replaced. This is a loss of £52,388 in invested funds. This fact, coupled with the fall in subscriptions to £5,438 from £7,838 in 1873, has caused much anxiety, and an appeal is being made to the residents in the neighbourhood who do not subscribe already. The Board wishes to correct the impression that the hospital is a rich one, not in need of support. Two additional beds have been endowed, making a total of ten.

Over £700 was paid in rates during the year, and efforts were made through the Central Hospital Council of London to obtain some revision of the rating of hospitals, but without success.

